# Lectures on Malformations of the Heart

**Published:** 1854-07

**Authors:** Thos. B. Peacock


*Lectures on Malformations of the Heart.* By THOS. B. PEACOCK, M.D.

*Cyanosis.*—There are few subjects in the range of Medical Science which have occasioned more discussion than the inquiry as to the immediate cause of the discoloration of the surface, which forms so marked a feature in most instances of malformation of the heart. Morgagni, in describing the case to which I have before referred, ascribed the marked cyanosis which had deen observed during life, to general congestion of the venous system, caused by the obstruction at the origin of the pulmonary artery. Dr. Hunter, on the contrary, seeing that in the case which he has related, the septum cordis was imperfect, so that the aorta was supplied from both ventricles, and that a large proportion of the blood circulating in the body must have been venous, supposed that the livid color the boy had presented during life, was owing to the intermixture of the currents of blood. These views have each since met with numerous supporters. The theory which ascribes the production of cyanosis chiefly to congestion of the venous system has been advocated by Ferrus and Louis in France, by Hasse and Rokanitsky in Germany, by Joy in this country, and very ably by Stillé in America. On the other hand, the view which refers the discoloration to the intermixture of the venous with the arterial blood, has, with various modifications, been supported by Gintrac and Bouillaud in France, by Meckel, in Germany, and by Farre, Paget, Williams and Hope. Corvisart and Laennec appear disposed to adopt the former explanation; and Dr. Chevers, while regarding the cyanosis as chiefly due to congestion, contends for the influence of the venous blood in the arteries, as modifying the intensity of the discoloration.

Gintrac, after a careful analysis of fifty-three cases of cyanosis, in all of which there was more or less intermixture of the currents of blood, inferred that the cyanosis was dependant on this cause, though he admitted that the intermixture was not always productive of cyanosis. Louis and Ferrus have dwelt more fully on the absence of any constant connection between the intermixture of the currents and the existence and intensity of cyanosis. Dr. Stillé, after a careful examination of a very extended series of cases of different forms of malformation of the heart, has shown that cyanosis may exist without the intermixture of the currents of blood; that there is no just proportion between the intensity of the cyanosis and the degree in which the blood is mixed; that complete intermixture of the blood may take place without cyanosis being produced; and that the variations in the extent, depth, and duration of the discoloration are inexplicable by the doctrine of the intermixture of blood. Of seventy-seven cases which he has collected and carefully analysed, he finds the condition of the pulmonary artery reported in sixty-two; and that in fifty-three of these it was contracted, obstructed, or impervious; while, in the remaining nine cases, there were other conditions present which would give rise to congestion of the venous system. He was therefore led to adopt the view of Morgagni and Louis, and to infer that cyanosis is dependant either on obstruction at the pulmonic orifice, or some other cause, giving rise to venous congestion. Congestion, he contends, not only satisfactorily accounts for the discoloration of the skin and the dyspnœa, but that it is always present when cyanosis exists, and is never found without the occurrence of cyanosis, unless there are satisfactory reasons for its absence.

In the previous lectures I have alluded to various cases which bear out the inferences of Dr. Stillé. I have mentioned the case of a girl in whom an abnormal septum was found in the right ventricle, without any other malformation of the heart, and who was markedly cyanotic during the several months she was under my observation, affording striking proofs that cyanosis may exist without mixture of the currents of blood. Cases of this kind are, however, much less frequent than those which display a want of just relation between the intensity of cyanosis and the amount of intermixture. In the other case of abnormal septum, which I have mentioned as having fallen under my own notice, you will remember that the aorta rose in great part from the right ventricle, so that a very large proportion of the blood circulating through the body must have been venous; yet there was no evidence that the boy had presented any material degree of cyanosis till shortly before his death; indeed, he had been an inmate of the Royal Free Hospital, for an accident, about twelve months before, and nothing unusual in his appearance had then been observed, the occurrence of cyanosis having apparently been manifested after the pulmonary artery became the subject of disease, by which its capacity was still further diminished. In the case of Dr. Hale, in which there existed only one ventricle; and that of Dr. G. A. Rees, in which the pulmonary artery gave off the descending aorta, not the slightest lividity was observed; so that these cases evince that the freest intermixture may exist without giving rise to cyanosis.

The fact that cyanosis is not always observed where abnormal communications exist between the two sides of the heart, has been admitted by the supporters of the theory of intermixture, and various reasons have been assigned for its absence; and especially it has been contended, that, provided the pressure on the two sides be equal, no intermixture will take place, though either septum be defective. To this it may, however, be answered, that the cyanotic symptoms are by no means always congenital, though the freest intermixture of the currents of blood must have existed from birth.

Lastly, cases frequently occur in which the variation in the degree of lividity cannot depend on any corresponding variation in the amount of intermixture. I recently saw an infant which suffered at intervals from the usual symptoms which attend malformations of the heart. While the child was quite quiet, there was no appearance of cyanosis, but the paroxysms were readily brought on by exposure to cold, and a loud systolic murmur was heard over the whole front of the chest, and most distinctly to the left side of the middle of the sternum. Under a mild alterative treatment, the paroxysms became less frequent, and had, indeed, ceased entirely when the child, then three months old, took hooping-cough, attended with bronchitis, when they recurred with much greater severity. On examination after death, the folds of the tricuspid valve were found somewhat adherent together, much thickened and indurated, and studded with recent fibrinous deposits; the right ventricle was hypertrophied and dilated, and the pulmonary artery of large size. At the base of the septum of the ventricles there existed two apertures, leading from the left ventricle, immediately below the origin of the aorta, into the sinus of the right ventricle. These apertures were larger on the left than on the right side, so that it was evident that the current of blood which had passed through them must have flowed from the left ventricle to the right; and, from their small size, they could neither have given passage to a large current, nor, from their hard and unyielding edges, could the quantity transmitted have been liable to material variation. The cyanosis could not, therefore, have been owing to the venous blood entering the left ventricle, and so being circulated through the body. Neither could the variations in its intensity have been due to any corresponding variation in the amount of intermixture. The different degrees of congestion of the venous system, consequent upon the increased difficulty in the transmission of blood through the lungs, could alone explain the recurrence of the paroxysms. In this case, also, the left arm and hand were at all times very livid, and somewhat swollen; and no other explanation of the fact was offered by examination, than that the venous trunks on that side had been compressed by enlarged glands at the root of the the lung and in their course.

In cases in which, notwithstanding that the intermixture of the currents of blood must always have existed, the cyanosis does not appear till comparatively late in life, the period of its accession may generally be traced to the occurrence of disease either in the heart or lungs, by which the original source of obstruction is aggravated; thus, during a slight attack of rheumatism, inflammation may attack valves previously malformed, so as to curtail still further the size of the opening into the pulmonary artery, or right ventricle; or the aperture may be so rigid and unyielding, as not to expand sufficiently to transmit the increased current of blood, required with the progress of growth; or an attack of bronchitis, by adding obstruction in the lungs, to that which already existed in the heart, may cause the aggravation of cyanosis, if previously present, or create it, where it had not before been observed.

From these considerations, we are, I think, correct in inferring, that the cyanosis is due to congestion of the venous system. I cannot however, concur in the opinion of Læennec, that the lividity in cases of malormation, differs in no degree from that which attends ordinary disease of the heart or lungs; and that, in some forms of affection of the lungs, the discoloration of the skin is as considerable, and as general as in cases of malformation. The cyanosis of malformation, when very marked, is much more intense than that from any other cause; but, occasionally, the lividity which attends pulmonary and cardiac disease is quite as intense as in some cases of malformation. In support of this I may instance the case of a boy, 17 years of age, who was a patient of mine at the

Royal Free Hospital in 1847, and had presented marked cyanosis from early llfe, yet in whom the discoloration was dependent on imperfect expansion of the lungs, connected with curvature of the spine, and the right ventricle was very greatly hypertrophied and dilated. That in cases of pulmonary and ordinary cardiac diseases the cyanosis is generally so much less intense that where the heart is malformed, must be ascribed to the amount of congestion being also less; for, in cases of acquired disease, were so small a proportion of blood submitted to the influence of the air, as in many cases of malformation, life could not be maintained. In cases of disease, also, the integuments generally become more or less œdematous, so that the lividity is masked.

Dr. Stillé's observations point too exclusively to contraction of the pulmonary orifice, as giving rise to the congestion on which the cyanosis is dependent. In the previous lectures, cases have been adduced in whieh the obstruction was caused by an abnormal septum in the right ventricle. An instance has just been mentioned in which it was dependent on the tricuspid valves; and, as before stated, it is sometimes caused by imperfect expansion of the lungs.

On the other hand, Dr. Chevers has shown, by reference to a case under the care of Dr. Lloyd, that great contraction of the orifice of the pulmonary artery, when it occurs in adult life, is not necessarily attended by any lividity. The case of Dr. Hamilton Roe, before mentioned. shows that cyanosis is not necessarily dependent on even great congenital contraction of the pulmonary orifice; while that described by Dr. Craigie, and one of my own, also, evince that the cyanosis, when present, does not always bear a strict relation to the amount of obstruction. In all exceptional cases of this kind, however, I believe it will be found that the right ventricle has acquired such an increase of power, as to enable it to overcome the difficulty in transmitting the blood through the contracted orifice. In my own case it is probable that the lividity had become less with the gradual diminution, during the progress of phthisis, of the amount of blood circulating in the body.

The inference to be drawn from the facts brought forward appears to be, that while obstruction to the flow of blood through tho lungs, or from or into the right ventricle, giving rise to general venous congestion, is the essential cause of cyanosis, the intensity of the lividity, and its peculiar color, are modified by other circumstances.

1st. It is probably necessary to the production of intense cyanosis, that, as suggested by Dr. Chevers, the obstruction to the circulation should either have been present before birth, when the capillary vessels are naturally more capacious than in the adult; or, that it should have existed before the full development of the body was attained, and while the entire vascular system was more readily dilatable; or, at least, that it should have been of long duration, so that the capillary vessels have become greatly expanded.

2d. The peculiar tint presented by persons laboring under malformation appears to depend in some degree on the condition of the integuments. Where the peculiar blue or black color is observed, the skin is usually very thin, and the body generally emaciated. Where the discoloration is rather of a deep rose tint, the patients are not much emaciated, or are even, in some cases, tolerably well nourished; and where the skin is pallid, there is either no material congestion, or it is masked by the œdematous condition of the integuments.

3d, and lastly. I cannot but think that the intensity and peculiar tint of the cyanosis must be much affected by the color of the blood in the vessels; so that where a very small proportion only is submitted to the influence of the air in the lungs, and the whole mass must therefore be of an unusually deep color, the general discoloration of the surface will be proportionally dark.—*London Med. Times.*

